# Transcriptional Reprogramming of Legume Genomes: Perspective and Challenges Associated With Single-Cell and Single Cell-Type Approaches During Nodule Development

**DOI:** 10.3389/fpls.2018.01600

**Published:** 2018-11-08

**Authors:** Marc Libault

**Affiliations:** ^1^Department of Agronomy and Horticulture, University of Nebraska-Lincoln, Lincoln, NE, United States; ^2^Centre for Plant Science Innovation, University of Nebraska-Lincoln, Lincoln, NE, United States; ^3^Center for Root and Rhizobiome Innovation, University of Nebraska-Lincoln, Lincoln, NE, United States

**Keywords:** legume, nodulation, root hair, single cell-type single-cell, transcriptome

## Abstract

Transcriptomic approaches revealed thousands of genes differentially or specifically expressed during nodulation, a biological process resulting from the symbiosis between leguminous plant roots and rhizobia, atmospheric nitrogen-fixing symbiotic bacteria. Ultimately, nodulation will lead to the development of a new root organ, the nodule. Through functional genomic studies, plant transcriptomes have been used by scientists to reveal plant genes potentially controlling nodulation. However, it is important to acknowledge that the physiology, transcriptomic programs, and biochemical properties of the plant cells involved in nodulation are continuously regulated. They also differ between the different cell-types composing the nodules. To generate a more accurate picture of the transcriptome, epigenome, proteome, and metabolome of the cells infected by rhizobia and cells composing the nodule, there is a need to implement plant single-cell and single cell-types strategies and methods. Accessing such information would allow a better understanding of the infection of plant cells by rhizobia and will help understanding the complex interactions existing between rhizobia and the plant cells. In this mini-review, we are reporting the current knowledge on legume nodulation gained by plant scientists at the level of single cell-types, and provide perspectives on single cell/single cell-type approaches when applied to legume nodulation.

## Introduction

Nodulation is a complex biological process which occurs between the root system of plants (i.e., legumes and the genus Parasponia of the *Ulmaceae* family) and rhizobia, soil bacteria capable to fix and assimilate the atmospheric dinitrogen. The establishment of nitrogen-fixing nodules requires two developmental programs, one leading to the formation of infection threads (plant-made structures through which rhizobia grow to reach the developing nodule) and one leading to nodule morphogenesis. Several molecular, physiological and cellular aspects of this biological interaction were characterized during the past two decades. For instance, the root and bacterial exudates used to initiate the recognition between the two partners are now well-characterized [e.g., plants flavonoids and iso-flavonoids (Phillips et al., 1994), bacterial nodulation factor (Nod factor) (Lerouge et al., 1990), and polysaccharides (Fraysse et al., 2003)]. More specifically, Nod factors are lipo-chitooligosaccharides whose synthesis is stimulated upon recognition of plant flavonoids by rhizobial NodD proteins (Oldroyd and Downie, 2008). Several functional genomic studies revealed the role of plant genes in controlling the perception then infection of the legume root hair cells and nodule cells by rhizobia (Oldroyd, 2013). Notably, the nodulation signaling pathway, a conserved gene regulatory pathway between legume species which is induced upon recognition of the Nod factor by Nod factor receptors, was characterized across several legume species (Oldroyd, 2013). In addition to these functional genomic studies, the development of microarrays followed by the emergence of high-throughput sequencing technologies led researchers to better characterize the overall response of the legume transcriptome to rhizobia inoculation and infection. For instance, these transcriptomic analyses were conducted to reveal the early responses of the legume root hair cells to rhizobia inoculation as well as the transcriptomic changes occurring during nodule development [see below for a more detailed description of these studies (Colebatch et al., 2002, 2004; Barnett et al., 2004; El Yahyaoui et al., 2004; Kouchi et al., 2004; Lee et al., 2004; Asamizu et al., 2005; Starker et al., 2006; Benedito et al., 2008; Brechenmacher et al., 2008; Hogslund et al., 2009; Libault et al., 2009; Afonso-Grunz et al., 2014; Roux et al., 2014; Kant et al., 2016; Peng et al., 2017; Yuan et al., 2017)].

While these studies allowed the identification of numerous differentially expressed genes, opening avenues for new functional analyses, the cellular complexity of the samples used to establish these transcriptomic resources remains a difficulty to accurately understand the response of plant cells to rhizobia inoculation and infection. For instance, only root hair cells localized in one specific zone of the root, the “susceptible zone” of the root system, are potentially infected. Similarly, only a subset of the nodule cells is infected by rhizobia upon endocytosis and formation of the symbiosome, a plant cell compartment containing the symbiotic bacteria. To overcome the problem associated with sample heterogeneity, researchers implemented strategies to isolate specific cell-types before applying the collection of high-throughput sequencing methodologies such as microarray hybridization and RNA-sequencing technology. Such strategy successfully revealed the activation and repression of transcriptomic programs in response to rhizobia inoculation and infection. In this mini-review, we are discussing the outcome of these analyses, their limitation, and opportunities to develop new strategies to better capture the dynamic changes of the legume transcriptome during the various stages of the nodulation process.

## Root Hair Infection by Rhizobia

The infection of the plant root hair cell by rhizobia is a continuous process which is initiated by the chemical recognition between plant and rhizobia [i.e., plants flavonoids and iso-flavonoids are recognized by the bacteria leading to the activation of the transcriptional regulators NodD (Fisher and Long, 1993), and bacterial Nod factors as well as exopolysaccharides are recognized by Lysin motif-receptor-like kinases of host plants (Limpens et al., 2003; Madsen et al., 2003; Radutoiu et al., 2003; Kawaharada et al., 2015)]. This recognition between the two partners is required to insure the specificity of the interaction and the success of the symbiosis. Upon recognition, the plant root hair cell will adopt molecular and morphological changes in order to enhance its infection by rhizobia. For instance, a gradual and constant reorientation of the direction of root hair growth will lead to the curling of the root hair cell. This curling is needed in order to trap rhizobia into an infection pocket to enhance the infection rate of the root hair cells. The reallocation of plasma membrane proteins in response to rhizobia is also one of the earliest responses of the plant to rhizobia inoculation. Specifically, several proteins of the microdomain fraction of the plasma membrane are reallocated at the tip of the root hair cells only several hours after bacterial inoculation (Haney and Long, 2010; Qiao et al., 2017). Functional analysis of the *Medicago truncatula* flotillin proteins suggest that this reallocation is needed before the formation of the pre-infection thread, then during the initiation and elongation of the infection thread and the progression of rhizobia in the root hair cells in this tubular structure (Haney and Long, 2010).

**FIGURE 1:**
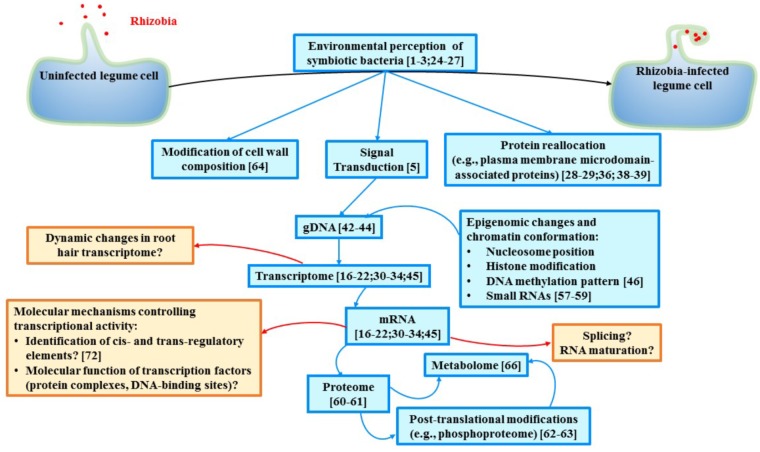
Schematic representation of the current biological knowledge gained during the past years in legume symbiosis at the level of single cell-types (blue boxes) and some of the major remaining gaps existing in our understanding of legume nodulation (red boxes). Relevant studies are mentioned in each box. In addition to gain more knowledge in various aspects of legume nodulation, data integration must be conducted. Ideally, multi-omic analyses at the level of single cell relevant to study the nodulation process (e.g., infected root hair cells and cells composing the nodule) should be conducted. Another challenge is related to the dynamic molecular changes occurring in those cells during the recognition, interaction, infection then symbiosis between plant cells and rhizobia. Taking in consideration the permanent adaptation of each cell involved in nodulation will clearly enhance our understanding of legume nodulation.

As described above, the initiation of the nodulation process results from sequential and progressive changes in root hair cell physiological, morphological, and molecular responses. While the morphological responses of the root hair cells consecutively to rhizobia inoculation (e.g., root hair cell branching and curling) are well-documented based on their ease to be monitored under the microscope, the molecular response of the root hair cells remains poorly described, especially when considering the specific programs required at each step of the infection of the root hair cell (El Yahyaoui et al., 2004; Kouchi et al., 2004; Lohar et al., 2006; Hogslund et al., 2009; Libault et al., 2010b; Breakspear et al., 2014; Damiani et al., 2016; Jardinaud et al., 2016; Figure 1). Having the objective to carefully decipher the transcriptomic programs and the time-course of gene activity consecutively to rhizobia inoculation, single cell-type strategies were implemented. For instance, researchers isolated root sections enriched in rhizobia-susceptible root hair cells (Lohar et al., 2006; Hogslund et al., 2009; Figure 1). This strategy was useful since it led to the identification of hundreds of genes differentially expressed in response to bacteria inoculation. To reach a higher level of resolution of these responses, populations of root hair cells were isolated from the root system at different time after bacterial inoculation (Libault et al., 2010b; Breakspear et al., 2014; Figure 1). Such approach highlighted the regulation of thousands of genes including many genes of the Nodulation Signaling Pathway, the differential expression of genes at different time of the infection, and the transient activation of the plant defense system (Libault et al., 2010b). The rapid inhibition of the plant defense system in root hair cells is likely required to promote the infection of the plant by rhizobia (El Yahyaoui et al., 2004; Kouchi et al., 2004; Libault et al., 2010b).

Despite this effort, the temporal regulation of the expression of the legume genes upon rhizobia inoculation was difficult to highlight. This is inherent to the nodulation process itself since new root hair cells are continuously infected and each infection is independently progressing from another. Hence, the lack of synchronization of the infection of the root hairs by rhizobia logically leads to the isolation of a heterogeneous plant material: a mixture of unresponsive root hair cells (those located outside of the susceptible zone of the root system), responsive but uninfected root hairs, and responsive and infected root hairs. The latter category could also be divided in unique populations of cells according to their stage of infection by rhizobia.

## The Legume Nodule, a Complex Root Organ

Concomitantly to root hair cell infection, the cortical cells of the root are actively dividing leading to the formation of the nodule primordia. The location of these divisions differ between legume species. For instance, in *M. truncatula*, the inner cortex and pericycle actively divide upon rhizobia inoculation whereas, in *Lotus japonicus*, the outer cortex cells divide (Szczyglowski et al., 1998; Timmers et al., 1999; Xiao et al., 2014). Alongside, the infection thread progresses in and between plant cells until it reaches those dividing cells. There, the bacteria which are differentiated in bacteroids, are released in the symbiosome, an organelle-like structure where the bacteroids are surrounded by the host plasma membrane. The presence of microdomain-associated proteins in the symbiosome membrane suggests a role of these membrane proteins in regulating the communication existing between the symbionts and the infected plant cells of the nodule (Haney and Long, 2010; Lefebvre et al., 2010; Qiao and Libault, 2017; Qiao et al., 2017).

Nodule organogenesis differs between legume species. For instance, indeterminate nodule development requires the maintenance of the primordia even upon formation of a mature indeterminate nodule (e.g., *M. truncatula* and *Pisum sativum*). Oppositely, in determinate nodules (e.g., *Glycine max*, *L. japonicus*, and *Phaseolus vulgaris*), the initially active nodule meristem will degenerate in mature nodules. As a consequence, the cellular organization differ between determinate and indeterminate nodules (Brewin, 1991; Ferguson et al., 2010). In indeterminate nodule four major zones can be distinguished. These zones are biologically different one from another. Zone #1 which is located at the tip of the nodule is the site of the permanent nodule meristem. Zone #2 corresponds to the infection zone where the bacteria infect the plant cells. Zone #3 is the nitrogen fixation zone where the bacteroids fix and assimilate for the plant the atmospheric dinitrogen. Zone #4 is located on the basal side of the nodule zone and is the location of the senescence of the nodule cells. Oppositely to indeterminate nodules, determinate nodules are not organized in zones. However, these nodules remain structurally organized: the plant cells colonized by rhizobia are exclusively located in the center of the globular nodules and are surrounded by uninfected epidermal, cortex, and vascular cells. In addition to their complex cellular composition, the nodules are also characterized by the level of endoreduplication of their cells, a duplication of the genomic DNA without cell division (Foucher and Kondorosi, 2000; Vinardell et al., 2003; Kondorosi and Kondorosi, 2004). While most plant cells contain 2C of genomic DNA, the infected cells of the nodules can reach 4, 8, 16, 32, 64C, etc., of genomic DNA content where C is the haploid DNA content. As a consequence, the zone #3 of indeterminate nodules is characterized by its massive endoreduplication.

To date, most transcriptomic analyses conducted on legume nodules focused on their developmental stages rather than their cellular complexity [e.g., *L. japonicus* (Colebatch et al., 2002, 2004; Kouchi et al., 2004; Asamizu et al., 2005; Hogslund et al., 2009), *M. truncatula* (Barnett et al., 2004; El Yahyaoui et al., 2004; Starker et al., 2006; Benedito et al., 2008), *G. max* (Lee et al., 2004; Brechenmacher et al., 2008; Libault et al., 2009; Yuan et al., 2017), *Cicer arietinum* (Afonso-Grunz et al., 2014; Kant et al., 2016), and *Arachis hypogaea* (Peng et al., 2017; Figure 1]. In indeterminate nodules, Roux et al. (2014) collected different zones of the *M. truncatula* nodules validating the use of laser microdissection in order to better depict the unique transcriptional properties of each zone. This method helps validating the used of laser microdissection to enhance the purity of the biological samples used from nodules (Roux et al., 2018). More recently, the same group revealed the role of Mt*DME* (*DEMETER*) as a major regulator of the transcriptional activity of nodule genes and transposable elements (Satge et al., 2016). However, additional biological information is needed to reveal the complexity of the transcriptional regulation, especially in determinate nodules.

## Applying Single-Cell/Single Cell-Type Approaches to Better Understand Legume Nodulation

In order to better understand the role of legume genes during the nodulation process, it is important to reveal the dynamic changes of their expression during nodulation (Figure 1). Such study should be conducted on infected root hair cells and nodule cells in order to capture the complexity of the molecular regulation at different stages of the infection of plant cells by the symbiotic bacteria. Accordingly, there is a need to isolate and separate each legume cell or cell-types (i.e., population of plant cells sharing the same biological function) infected by rhizobia or contributing to nodulation such as the root hairs preferentially located in the susceptible zone of the root and the different nodule cell-types (e.g., epidermal cells, vascular cells, and infected and uninfected cortex cells of the nodule).

Various methodologies were established to isolate plant cell-types (see Libault et al., 2017 for review). These methods consist in isolating transgenic plant cell protoplasts (i.e., living plant cells devoid in cell walls upon digestion of the cell wall by a cocktail of cellulases, hemicellulases, and pectinases) expressing the green fluorescent protein (GFP) in a cell-type dependent manner using fluorescent-activated cell sorting (FACS) (Birnbaum et al., 2003; Brady et al., 2007; Dinneny et al., 2008; Iyer-Pascuzzi et al., 2011; Petersson et al., 2015; Marx, 2016). Another approach consists in sequencing the transcriptome of cell nuclei upon their isolation (e.g., isolation of biotinylated nuclei) expecting that the cellular and nuclear transcriptomes are similar (Deal and Henikoff, 2011). A more sophisticated approach allowing the sequencing of transcripts interacting with ribosomes consists in the isolation of mRNA using a cell type-preferential tagged ribosomal protein (Zanetti et al., 2005). Applying those methods, genes preferentially expressed in specific root cell-types were characterized validating the idea of root cell-type-preferential transcriptomes. More recently, the gDNA methylation profiles from 6 different root cell-types from Arabidopsis were established (Kawakatsu et al., 2016). Another strategy successfully applied when analyzing the transcriptomic, epigenomic (Yan et al., 2013, 2015, 2016), proteomic (Larrainzar et al., 2007; Thal et al., 2018), phosphoproteomic (Nguyen et al., 2012; Rose et al., 2012), metabolomics (Brechenmacher et al., 2010), and glycomic (Muszynski et al., 2015) responses of legume plants during the nodulation process consist in to the massive isolation of root hairs inoculated with rhizobia (Brechenmacher et al., 2009, 2012; Libault et al., 2010a,c).

However, single cell-type approaches have several limitations when considering the nodulation process. For instance, while the isolation of a population of legume root hairs enhances plant sample homogeneity leading to a more accurate depiction of the molecular mechanisms controlling root hair infection by rhizobia, it is important to acknowledge the heterogeneity of this cellular population according to their unique stages of differentiation, unique responses to their environment, different stages in their infection by rhizobia, and the stochastic variations existing between cells. Also, other strategies need to be established to properly investigate the unique transcriptomic signature of the cells composing the nodule. To overcome these limitations, single-cell approaches (i.e., individual analysis of the transcriptome of each cell composing a complex organ) coupled with droplet-based microfluidic systems (Kolodziejczyk et al., 2015) are emerging. These systems [e.g., Chromium Single Cell Gene Expression Solution (10× Genomics), ddSEq (Bio-Rad), C1 (Fluidigm)] allow the separation and isolation of each single-cell preliminary to their molecular analysis. However, there are several technical limitations to consider when using these droplet-based microfluidic systems. For instance, the use of plant protoplasts in droplet-based microfluidic systems remains challenging due to the cell size discrimination of these systems (e.g., the 10× Genomics gel beads and C1 Fluidigm chips cannot incorporate cells/nuclei larger than 52 and 25 μm of diameter, respectively). This size exclusion might lead to the absence or relative depletion of the transcriptome of large plant cells. Also, protoplast bursting remains a major concern leading to a decrease in RNA-seq library construction efficiency and a marginal representation of low-represented cell types (Shulse et al., 2018). Consequently, isolated plant nuclei represent an interesting alternative but it presupposes that the cell and nuclear transcriptomes are similar. Previous studies concluded that working on isolated nuclei is an acceptable way to overcome the problem of fragile cells (Deal and Henikoff, 2011). There is a need to validate this results on plant cells before to fully consider isolated nuclei as an alternative to single-cell biology. Consequently, the application of droplet technology on plant cells will require the combination of unique expertise in plant cell biology, molecular biology, and bioinformatics in order to generate viable biological samples compatible with droplet-based microfluidic systems. Being capable to overcome these limitations will open new avenues not only to understand legume nodulation but also to reveal the dynamic changes of the plant cell molecular responses during the infection process (Figure 1).

## Conclusion and Perspectives

Accessing single-cell transcriptomes is only a first step to fully understand legume nodulation. Additional avenues must be considered in order to develop a system-level understanding of legume nodulation including the integration of transcriptomic, epigenomic, proteomic, and metabolomics datasets. In addition, gene regulatory networks including the characterization of the binding sites of transcription factors controlling the nodulation process (Andriankaja et al., 2007) should also be more systematically characterized. As mentioned above, such experiments should be conducted at the level of single cells or, at least, at the level of single cell-types. In order to reach this goal, new strategies and technologies has been recently applied on plants or should be adapted to plant single cell biology (Figure 1). For instance, recent improvements of the sensitivity of mass-spectrometers and the development of new biochemical tools allow the characterization of plant single-cell proteomes (Misra et al., 2014; Zhu et al., 2016), and the three-dimensional spatial distributions of plant metabolites including from soybean nodules (Stopka et al., 2017; Velickovic et al., 2018). The establishment of single cell ATAC-seq methodology [Assay for Transposase-Accessible Chromatin using sequencing (Cusanovich et al., 2015)] to reveal the folding of the chromatin fiber of eukaryotic cells at the level of single cell also represents an interesting approach to better understand the impact of the epigenome on gene expression. However, the future access to such methodology will need to be adapted and applied to plant single cells.

## Author Contributions

ML designed, wrote, and edited this mini-review.

## Conflict of Interest Statement

The author declares that the research was conducted in the absence of any commercial or financial relationships that could be construed as a potential conflict of interest.

## References

[B1] Afonso-Grunz F. Molina C. Hoffmeier K. Rycak L. Kudapa H. Varshney R. K. (2014). Genome-based analysis of the transcriptome from mature chickpea root nodules. *Front. Plant Sci.* 5:325. 10.3389/fpls.2014.00325 25071808 PMC4093793

[B2] Andriankaja A. Boisson-Dernier A. Frances L. Sauviac L. Jauneau A. Barker D. G. (2007). AP2-ERF transcription factors mediate Nod factor dependent Mt ENOD11 activation in root hairs via a novel cis-regulatory motif. *Plant Cell* 19 2866–2885. 10.1105/tpc.107.052944 17827349 PMC2048698

[B3] Asamizu E. Nakamura Y. Sato S. Tabata S. (2005). Comparison of the transcript profiles from the root and the nodulating root of the model legume *Lotus japonicus* by serial analysis of gene expression. *Mol. Plant Microbe Interact.* 18 487–498. 10.1094/MPMI-18-0487 15915647

[B4] Barnett M. J. Toman C. J. Fisher R. F. Long S. R. (2004). A dual-genome symbiosis chip for coordinate study of signal exchange and development in a prokaryote-host interaction. *Proc. Natl. Acad. Sci. U.S.A.* 101 16636–16641. 10.1073/pnas.0407269101 15542588 PMC527922

[B5] Benedito V. A. Torres-Jerez I. Murray J. D. Andriankaja A. Allen S. Kakar K. (2008). A gene expression atlas of the model legume *Medicago truncatula.* *Plant J.* 55 504–513. 10.1111/j.1365-313X.2008.03519.x 18410479

[B6] Birnbaum K. Shasha D. E. Wang J. Y. Jung J. W. Lambert G. M. Galbraith D. W. (2003). A gene expression map of the Arabidopsis root. *Science* 302 1956–1960. 10.1126/science.1090022 14671301

[B7] Brady S. M. Orlando D. A. Lee J. Y. Wang J. Y. Koch J. Dinneny J. R. (2007). A high-resolution root spatiotemporal map reveals dominant expression patterns. *Science* 318 801–806. 10.1126/science.1146265 17975066

[B8] Breakspear A. Liu C. Roy S. Stacey N. Rogers C. Trick M. (2014). The root hair “infectome” of *Medicago truncatula* uncovers changes in cell cycle genes and reveals a requirement for Auxin signaling in rhizobial infection. *Plant Cell* 26 4680–4701. 10.1105/tpc.114.133496 25527707 PMC4311213

[B9] Brechenmacher L. Kim M. Y. Benitez M. Li M. Joshi T. Calla B. (2008). Transcription profiling of soybean nodulation by *Bradyrhizobium japonicum*. *Mol. Plant Microbe Interact.* 21 631–645. 10.1094/MPMI-21-5-0631 18393623

[B10] Brechenmacher L. Lee J. Sachdev S. Song Z. Nguyen T. H. Joshi T. (2009). Establishment of a protein reference map for soybean root hair cells. *Plant Physiol.* 149 670–682. 10.1104/pp.108.131649 19036831 PMC2633823

[B11] Brechenmacher L. Lei Z. Libault M. Findley S. Sugawara M. Sadowsky M. J. (2010). Soybean metabolites regulated in root hairs in response to the symbiotic bacterium *Bradyrhizobium japonicum*. *Plant Physiol.* 153 1808–1822. 10.1104/pp.110.157800 20534735 PMC2923908

[B12] Brechenmacher L. Nguyen T. H. Hixson K. Libault M. Aldrich J. Pasa-Tolic L. (2012). Identification of soybean proteins from a single cell type: the root hair. *Proteomics* 12 3365–3373. 10.1002/pmic.201200160 22997094

[B13] Brewin N. J. (1991). Development of the legume root nodule. *Annu. Rev. Cell Biol.* 7 191–226. 10.1146/annurev.cb.07.110191.001203 1809347

[B14] Colebatch G. Desbrosses G. Ott T. Krusell L. Montanari O. Kloska S. (2004). Global changes in transcription orchestrate metabolic differentiation during symbiotic nitrogen fixation in *Lotus japonicus*. *Plant J.* 39 487–512. 10.1111/j.1365-313X.2004.02150.x 15272870

[B15] Colebatch G. Kloska S. Trevaskis B. Freund S. Altmann T. Udvardi M. K. (2002). Novel aspects of symbiotic nitrogen fixation uncovered by transcript profiling with cDNA arrays. *Mol. Plant Microbe Interact.* 15 411–420. 10.1094/MPMI.2002.15.5.411 12036271

[B16] Cusanovich D. A. Daza R. Adey A. Pliner H. A. Christiansen L. Gunderson K. L. (2015). Multiplex single cell profiling of chromatin accessibility by combinatorial cellular indexing. *Science* 348 910–914. 10.1126/science.aab1601 25953818 PMC4836442

[B17] Damiani I. Drain A. Guichard M. Balzergue S. Boscari A. Boyer J. C. (2016). Nod factor effects on root hair-specific transcriptome of *Medicago truncatula*: focus on plasma membrane transport systems and reactive oxygen species networks. *Front. Plant Sci.* 7:794. 10.3389/fpls.2016.00794 27375649 PMC4894911

[B18] Deal R. B. Henikoff S. (2011). The INTACT method for cell type-specific gene expression and chromatin profiling in *Arabidopsis thaliana*. *Nat. Protoc.* 6 56–68. 10.1038/nprot.2010.175 21212783 PMC7219316

[B19] Dinneny J. R. Long T. A. Wang J. Y. Jung J. W. Mace D. Pointer S. (2008). Cell identity mediates the response of Arabidopsis roots to abiotic stress. *Science* 320 942–945. 10.1126/science.1153795 18436742

[B20] El Yahyaoui F. Kuster H. Ben Amor B. Hohnjec N. Puhler A. Becker A. (2004). Expression profiling in *Medicago truncatula* identifies more than 750 genes differentially expressed during nodulation, including many potential regulators of the symbiotic program. *Plant Physiol.* 136 3159–3176. 10.1104/pp.104.043612 15466239 PMC523376

[B21] Ferguson B. J. Indrasumunar A. Hayashi S. Lin M. H. Lin Y. H. Reid D. E. (2010). Molecular analysis of legume nodule development and autoregulation. *J. Integr. Plant Biol.* 52 61–76. 10.1111/j.1744-7909.2010.00899.x 20074141

[B22] Fisher R. F. Long S. R. (1993). Interactions of Nodd at the Nod box - Nodd binds to 2 distinct sites on the same face of the helix and induces a bend in the DNA. *J. Mol. Biol.* 233 336–348. 10.1006/jmbi.1993.1515 8411148

[B23] Foucher F. Kondorosi E. (2000). Cell cycle regulation in the course of nodule organogenesis in *Medicago*. *Plant Mol. Biol.* 43 773–786. 10.1023/A:1006405029600 11089876

[B24] Fraysse N. Couderc F. Poinsot V. (2003). Surface polysaccharide involvement in establishing the rhizobium-legume symbiosis. *Eur. J. Biochem.* 270 1365–1380. 10.1046/j.1432-1033.2003.03492.x 12653992

[B25] Haney C. H. Long S. R. (2010). Plant flotillins are required for infection by nitrogen-fixing bacteria. *Proc. Natl. Acad. Sci. U.S.A.* 107 478–483. 10.1073/pnas.0910081107 20018678 PMC2806772

[B26] Hogslund N. Radutoiu S. Krusell L. Voroshilova V. Hannah M. A. Goffard N. (2009). Dissection of symbiosis and organ development by integrated transcriptome analysis of *Lotus japonicus* mutant and wild-type plants. *PLoS One* 4:e6556. 10.1371/journal.pone.0006556 19662091 PMC2717213

[B27] Iyer-Pascuzzi A. S. Jackson T. Cui H. Petricka J. J. Busch W. Tsukagoshi H. (2011). Cell identity regulators link development and stress responses in the Arabidopsis root. *Dev. Cell* 21 770–782. 10.1016/j.devcel.2011.09.009 22014526 PMC3204215

[B28] Jardinaud M. F. Boivin S. Rodde N. Catrice O. Kisiala A. Lepage A. (2016). A laser dissection-RNAseq analysis highlights the activation of cytokinin pathways by Nod factors in the *Medicago truncatula* root epidermis. *Plant Physiol.* 171 2256–2276. 10.1104/pp.16.00711 27217496 PMC4936592

[B29] Kant C. Pradhan S. Bhatia S. (2016). Dissecting the root nodule transcriptome of chickpea (*Cicer arietinum* L.). *PLoS One* 11:e0157908. 10.1371/journal.pone.0157908 27348121 PMC4922567

[B30] Kawaharada Y. Kelly S. Nielsen M. W. Hjuler C. T. Gysel K. Muszynski A. (2015). Receptor-mediated exopolysaccharide perception controls bacterial infection. *Nature* 523 308–312. 10.1038/nature14611 26153863

[B31] Kawakatsu T. Stuart T. Valdes M. Breakfield N. Schmitz R. J. Nery J. R. (2016). Unique cell-type-specific patterns of DNA methylation in the root meristem. *Nat. Plants* 2:16058. 10.1038/nplants.2016.58 27243651 PMC4855458

[B32] Kolodziejczyk A. A. Kim J. K. Svensson V. Marioni J. C. Teichmann S. A. (2015). The technology and biology of single-cell RNA sequencing. *Mol. Cell* 58 610–620. 10.1016/j.molcel.2015.04.005 26000846

[B33] Kondorosi E. Kondorosi A. (2004). Endoreduplication and activation of the anaphase-promoting complex during symbiotic cell development. *FEBS Lett.* 567 152–157. 10.1016/j.febslet.2004.04.075 15165909

[B34] Kouchi H. Shimomura K. Hata S. Hirota A. Wu G. J. Kumagai H. (2004). Large-scale analysis of gene expression profiles during early stages of root nodule formation in a model legume, *Lotus japonicus*. *DNA Res.* 11 263–274. 10.1093/dnares/11.4.263 15500251

[B35] Larrainzar E. Wienkoop S. Weckwerth W. Ladrera R. Arrese-Igor C. Gonzalez E. M. (2007). *Medicago truncatula* root nodule proteome analysis reveals differential plant and bacteroid responses to drought stress. *Plant Physiol.* 144 1495–1507. 10.1104/pp.107.101618 17545507 PMC1914115

[B36] Lee H. Hur C. G. Oh C. J. Kim H. B. Pakr S. Y. An C. S. (2004). Analysis of the root nodule-enhanced transcriptome in soybean. *Mol. Cells* 18 53–62. 15359124

[B37] Lefebvre B. Timmers T. Mbengue M. Moreau S. Herve C. Toth K. (2010). A remorin protein interacts with symbiotic receptors and regulates bacterial infection. *Proc. Natl. Acad. Sci. U.S.A.* 107 2343–2348. 10.1073/pnas.0913320107 20133878 PMC2836688

[B38] Lerouge P. Roche P. Faucher C. Maillet F. Truchet G. Prome J. C. (1990). Symbiotic host-specificity of *Rhizobium meliloti* is determined by a sulphated and acylated glucosamine oligosaccharide signal. *Nature* 344 781–784. 10.1038/344781a0 2330031

[B39] Libault M. Brechenmacher L. Cheng J. Xu D. Stacey G. (2010a). Root hair systems biology. *Trends Plant Sci.* 15 641–650. 10.1016/j.tplants.2010.08.010 20851035

[B40] Libault M. Farmer A. Brechenmacher L. Drnevich J. Langley R. J. Bilgin D. D. (2010b). Complete transcriptome of the soybean root hair cell, a single-cell model, and its alteration in response to *Bradyrhizobium japonicum* infection. *Plant Physiol.* 152 541–552. 10.1104/pp.109.148379 19933387 PMC2815892

[B41] Libault M. Farmer A. Brechenmacher L. May G. D. Stacey G. (2010c). Soybean root hairs: a valuable system to investigate plant biology at the cellular level. *Plant Signal. Behav.* 5 419–421. 20339317 10.4161/psb.5.4.11283 PMC7080419

[B42] Libault M. Joshi T. Takahashi K. Hurley-Sommer A. Puricelli K. Blake S. (2009). Large-scale analysis of putative soybean regulatory gene expression identifies a Myb gene involved in soybean nodule development. *Plant Physiol.* 151 1207–1220. 10.1104/pp.109.144030 19755542 PMC2773063

[B43] Libault M. Pingault L. Zogli P. Schiefelbein J. (2017). Plant systems biology at the single-cell level. *Trends Plant Sci.* 22 949–960. 10.1016/j.tplants.2017.08.006 28970001

[B44] Limpens E. Franken C. Smit P. Willemse J. Bisseling T. Geurts R. (2003). LysM domain receptor kinases regulating rhizobial Nod factor-induced infection. *Science* 302 630–633. 10.1126/science.1090074 12947035

[B45] Lohar D. P. Sharopova N. Endre G. Penuela S. Samac D. Town C. (2006). Transcript analysis of early nodulation events in *Medicago truncatula.* *Plant Physiol.* 140 221–234. 10.1104/pp.105.070326 16377745 PMC1326046

[B46] Madsen E. B. Madsen L. H. Radutoiu S. Olbryt M. Rakwalska M. Szczyglowski K. (2003). A receptor kinase gene of the LysM type is involved in legume perception of rhizobial signals. *Nature* 425 637–640. 10.1038/nature02045 14534591

[B47] Marx V. (2016). Plants: a tool box of cell-based assays. *Nat. Methods* 13 551–554. 10.1038/nmeth.3900 27355791

[B48] Misra B. B. Assmann S. M. Chen S. (2014). Plant single-cell and single-cell-type metabolomics. *Trends Plant Sci.* 19 637–646. 10.1016/j.tplants.2014.05.005 24946988

[B49] Muszynski A. O’Neill M. A. Ramasamy E. Pattathil S. Avci U. Pena M. J. (2015). Xyloglucan, galactomannan, glucuronoxylan, and rhamnogalacturonan I do not have identical structures in soybean root and root hair cell walls. *Planta* 242 1123–1138. 10.1007/s00425-015-2344-y 26067758

[B50] Nguyen T. H. Brechenmacher L. Aldrich J. T. Clauss T. R. Gritsenko M. A. Hixson K. K. (2012). Quantitative phosphoproteomic analysis of soybean root hairs inoculated with *Bradyrhizobium japonicum*. *Mol. Cell. Proteomics* 11 1140–1155. 10.1074/mcp.M112.018028 22843990 PMC3494206

[B51] Oldroyd G. E. (2013). Speak, friend, and enter: signalling systems that promote beneficial symbiotic associations in plants. *Nat. Rev. Microbiol.* 11 252–263. 10.1038/nrmicro2990 23493145

[B52] Oldroyd G. E. Downie J. A. (2008). Coordinating nodule morphogenesis with rhizobial infection in legumes. *Annu. Rev. Plant Biol.* 59 519–546. 10.1146/annurev.arplant.59.032607.092839 18444906

[B53] Peng Z. Liu F. Wang L. Zhou H. Paudel D. Tan L. (2017). Transcriptome profiles reveal gene regulation of peanut (*Arachis hypogaea* L.) nodulation. *Sci. Rep.* 7:40066. 10.1038/srep40066 28059169 PMC5216375

[B54] Petersson S. V. Linden P. Moritz T. Ljung K. (2015). Cell-type specific metabolic profiling of *Arabidopsis thaliana* protoplasts as a tool for plant systems biology. *Metabolomics* 11 1679–1689. 10.1007/s11306-015-0814-7 26491421 PMC4605972

[B55] Phillips D. A. Dakora F. D. Sande E. Joseph C. M. Zon J. (1994). Synthesis, release, and transmission of alfalfa signals to rhizobial symbionts. *Plant Soil* 161 69–80. 10.1007/BF02183086

[B56] Qiao Z. Brechenmacher L. Smith B. Strout G. W. Mangin W. Taylor C. (2017). The GmFWL1 (FW2-2-like) nodulation gene encodes a plasma membrane microdomain-associated protein. *Plant Cell Environ.* 40 1442–1455. 10.1111/pce.12941 28241097

[B57] Qiao Z. Libault M. (2017). Function of plasma membrane microdomain-associated proteins during legume nodulation. *Plant Signal. Behav.* 12 e1365215. 10.1080/15592324.2017.1365215 28816608 PMC5647967

[B58] Radutoiu S. Madsen L. H. Madsen E. B. Felle H. H. Umehara Y. Gronlund M. (2003). Plant recognition of symbiotic bacteria requires two LysM receptor-like kinases. *Nature* 425 585–592. 10.1038/nature02039 14534578

[B59] Rose C. M. Venkateshwaran M. Volkening J. D. Grimsrud P. A. Maeda J. Bailey D. J. (2012). Rapid phosphoproteomic and transcriptomic changes in the rhizobia-legume symbiosis. *Mol. Cell. Proteomics* 11 724–744. 10.1074/mcp.M112.019208 22683509 PMC3434772

[B60] Roux B. Rodde N. Jardinaud M. F. Timmers T. Sauviac L. Cottret L. (2014). An integrated analysis of plant and bacterial gene expression in symbiotic root nodules using laser-capture microdissection coupled to RNA sequencing. *Plant J.* 77 817–837. 10.1111/tpj.12442 24483147

[B61] Roux B. Rodde N. Moreau S. Jardinaud M. F. Gamas P. (2018). Laser capture micro-dissection coupled to RNA sequencing: a powerful approach applied to the model legume *Medicago truncatula* in interaction with *Sinorhizobium meliloti*. *Methods Mol. Biol.* 1830 191–224. 10.1007/978-1-4939-8657-6_12 30043372

[B62] Satge C. Moreau S. Sallet E. Lefort G. Auriac M. C. Rembliere C. (2016). Reprogramming of DNA methylation is critical for nodule development in *Medicago truncatula.* *Nat. Plants* 2:16166. 10.1038/nplants.2016.166 27797357

[B63] Shulse C. C. B. Turco G. Zhu Y. Brady S. Dickel D. (2018). High-throughput single-cell transcriptome profiling of plant cell types. *bioRxiv* [Preprint]. 10.1101/402966 PMC6758921 31091459

[B64] Starker C. G. Parra-Colmenares A. L. Smith L. Mitra R. M. Long S. R. (2006). Nitrogen fixation mutants of *Medicago truncatula* fail to support plant and bacterial symbiotic gene expression. *Plant Physiol.* 140 671–680. 10.1104/pp.105.072132 16407449 PMC1361333

[B65] Stopka S. A. Agtuca B. J. Koppenaal D. W. Pasa-Tolic L. Stacey G. Vertes A. (2017). Laser-ablation electrospray ionization mass spectrometry with ion mobility separation reveals metabolites in the symbiotic interactions of soybean roots and rhizobia. *Plant J.* 91 340–354. 10.1111/tpj.13569 28394446

[B66] Szczyglowski K. Shaw R. S. Wopereis J. Copeland S. Hamburger D. Kasiborski B. (1998). Nodule organogenesis and symbiotic mutants of the model legume *Lotus japonicus*. *Mol. Plant Microbe Interact.* 11 684–697. 10.1094/MPMI.1998.11.7.684

[B67] Thal B. Braun H. P. Eubel H. (2018). Proteomic analysis dissects the impact of nodulation and biological nitrogen fixation on Vicia faba root nodule physiology. *Plant Mol. Biol.* 97 233–251. 10.1007/s11103-018-0736-7 29779088

[B68] Timmers A. C. J. Auriac M. C. Truchet G. (1999). Refined analysis of early symbiotic steps of the *Rhizobium*-*Medicago* interaction in relationship with microtubular cytoskeleton rearrangements. *Development* 126 3617–3628. 10409507 10.1242/dev.126.16.3617

[B69] Velickovic D. Agtuca B. J. Stopka S. A. Vertes A. Koppenaal D. W. Pasa-Tolic L. (2018). Observed metabolic asymmetry within soybean root nodules reflects unexpected complexity in rhizobacteria-legume metabolite exchange. *ISME J.* 12 2335–2338. 10.1038/s41396-018-0188-8 29899508 PMC6092352

[B70] Vinardell J. M. Fedorova E. Cebolla A. Kevei Z. Horvath G. Kelemen Z. (2003). Endoreduplication mediated by the anaphase-promoting complex activator CCS52A is required for symbiotic cell differentiation in *Medicago truncatula* nodules. *Plant Cell* 15 2093–2105. 10.1105/tpc.014373 12953113 PMC181333

[B71] Xiao T. T. Schilderink S. Moling S. Deinum E. E. Kondorosi E. Franssen H. (2014). Fate map of *Medicago truncatula* root nodules. *Development* 141 3517–3528. 10.1242/dev.110775 25183870

[B72] Yan Z. Hossain M. S. Arikit S. Valdes-Lopez O. Zhai J. Wang J. (2015). Identification of microRNAs and their mRNA targets during soybean nodule development: functional analysis of the role of miR393j-3p in soybean nodulation. *New Phytol.* 207 748–759. 10.1111/nph.13365 25783944

[B73] Yan Z. Hossain M. S. Valdes-Lopez O. Hoang N. T. Zhai J. Wang J. (2016). Identification and functional characterization of soybean root hair microRNAs expressed in response to *Bradyrhizobium japonicum* infection. *Plant Biotechnol. J.* 14 332–341. 10.1111/pbi.12387 25973713 PMC11388829

[B74] Yan Z. Hossain M. S. Wang J. Valdes-Lopez O. Liang Y. Libault M. (2013). miR172 regulates soybean nodulation. *Mol. Plant Microbe Interact.* 26 1371–1377. 10.1094/MPMI-04-13-0111-R 23980625

[B75] Yuan S. L. Li R. Chen H. F. Zhang C. J. Chen L. M. Hao Q. N. (2017). RNA-Seq analysis of nodule development at five different developmental stages of soybean (Glycine max) inoculated with *Bradyrhizobium japonicum* strain 113-2. *Sci. Rep.* 7:42248. 10.1038/srep42248 28169364 PMC5294573

[B76] Zanetti M. E. Chang I. F. Gong F. Galbraith D. W. Bailey-Serres J. (2005). Immunopurification of polyribosomal complexes of Arabidopsis for global analysis of gene expression. *Plant Physiol.* 138 624–635. 10.1104/pp.105.059477 15955926 PMC1150383

[B77] Zhu Y. Li H. Bhatti S. Zhou S. Yang Y. Fish T. (2016). Development of a laser capture microscope-based single-cell-type proteomics tool for studying proteomes of individual cell layers of plant roots. *Hortic. Res.* 3:16026. 10.1038/hortres.2016.26 27280026 PMC4888759

